# Importance of Proper Alimentation in Health and Disorders

**Published:** 1870-11

**Authors:** Augustus Trafton


*Importance of Proper Alimentation in Health and Disease.*— Read before the Sacramento Medical Society, by AUGUSTUS TRAFTON, M.D.

In attempting to elucidate the importance of alimentation in its various relations to the human body, I feel a diffidence that is inspired by a consciousness of my inability to do justice to so important a subject. I shall therefore beg the indulgence of the Society while I read these few hasty and incomplete notes; not so much with a hope of instructing, as a desire to awaken attention and provoke discussion on this important subject. Aliments may be defined as all substances which are absolutely required to repair the waste of the animal body, to supply materials for its growth, and to maintain all the various functions at a healthy standard. That the power of self-regeneration is one of the most distinctive properties of the human body, is a fact evident to the most careless observer. This regeneration consists in the incessant molecular change incident to and dependent on the absorption and assimilation of aliment of various kinds. Its activity may be checked or hastened by various conditions of the system, but it cannot be completely arrested during the continuance of life. In well-nourished individuals the blood contains all the elements necessary for the regeneration of the various tissues of the body; but owing to the constant physiological decay of the organism the blood soon becomes impoverished, and it is necessary to supply the proper material for a new supply of blood, which process involves the ingestion of various articles of food and drink.

In health the wants of the system are made known to us by a sensation of hunger or thirst. Hunger probably has its origin in the stomach, and is transmitted to the brain probably by the pneumogastric nerve: I say probably, because it is not certain that the sensation is produced in the stomach, but it may be a general feeling of want produced by the lack of material in the blood to supply the waste of the system; and we refer it to the stomach, because of its proximity to the heart and large blood-vessels. It has also been demonstrated by at least one observer that section of the pneumogastric nerve does not destroy or abolish the desire for food.

The demand of the system for water is much more urgent and annoying than the desire for food. This sensation, unlike hunger, is never agreeable, and when prolonged abstinence from drink is practiced, the thirst becomes painful in the extreme. The blood becomes thicker, and all the secretions are diminished. Death takes place much sooner in consequence of the privation of fluids than where only solid aliments are denied.

*Inanition* is that condition of the system produced by total or partial privation of proper aliments. If food be improper in quality or insufficient in quantity to nourish the system, the same effect is produced; or if the organs proper for the digestion and assimilation of food be in any way prevented from performing their normal functions, the regeneration of the blood is prevented and the tissues are starved in consequence, although food may have been taken into the stomach and passed through the alimentary canal. One of the first and most notable consequences of inanition is a *loss of weight;* the body loses its fair proportions day by day in a regular, progressive manner, long after the process of reparation has ceased in most of the organs.

Death occurs when a certain point is reached, with a great deal of regularity; ordinarily occurring when the body loses about four-tenths or five-tenths of its normal weight. The fatty parts are first affected, and completely disappear. According to a table prepared by Chossat, the fat, blood, spleen, pancreas, liver, heart, intestines, and muscles of locomotion, lose more than four-tenths of their weight before death takes place. The following organs lose in the order in which they are named, less than fourtenths at the period of dissolution, viz.: stomach, pharynx, and esophagus, skin, kidneys, respiratory apparatus, osseous system, eyes, and nervous system. The brain is probably the last organ to suffer, and loses the least in weight.

Temperature, age, sex, exercise, light, moisture, and the passions, all exert an influence more or less marked on the process of nutrition, increasing or retarding, as the case may be, the development of the tissues of the human body. The blood changes its appearance during the progress of starvation most notably; it is impoverished to an extreme degree, and finally refuses to circulate in the capillary vessels of the skin and some other tissues. The heart's action is sensibly diminished in power and frequency, as a rule, in proportion to the diminution of the temperature of the body; being reduced to thirty or forty beats per minute when the temperature has reached 75° or 80° Fahr. The amount of carbonic acid exhaled is diminished, and the number of respirations is generally decreased, while the breath becomes extremely fetid; the muscular movements slow and uncertain; the voice weakens to a whisper, and memory resigns her seat.

These are some of the phenomena of inanition, occurring in a healthy subject. If such be the case, the neglect of proper alimentation, when the body is prostrated by disease, can but be greatly injurious. Insufficient alimentation during disease is a cause of death, which is often overlooked because of its silent and insidious advances; the patient having no desire for food (owing to his blunted and imperfect sensibility), often neglects or refuses to take sufficient nourishment, and finally succumbs—not so much to the disease as to the lack of proper aliment. Too much food, although injurious, is generally tolerated by the system, and disposed of through some of the excretory organs; but too little food produces a condition that can never be remedied, the system having no power to make up a deficiency. Whenever the waste of the body goes beyond the supply, death is the inevitable consequence. It may be slower, but not less sure, than when all supplies of food are cut off.

Having shown that death takes place from inanition when we deprive the human subject of a proper quantity of nutritious aliment, it will not require much argument to show that persons not in a healthy condition must have proper food in sufficient quantities, not only to enable them to live, but to be able to resist the tendency to death produced by the malady or maladies with which they are afflicted.

I have already defined *alimentary substances* to be such as are absolutely required to repair the waste of the body, to supply materials for its growth, and to maintain all the functions of that body at a healthy standard. In this list are included articles from the animal, vegetable, and inorganic kingdoms, susceptible of division and subdivision into many classes: the most important are organic, nitrogenized principles from both animal and vegetable kingdoms, viz., albumen, fibrin, musculine, caseine, gelatine, gluten, legumine, etc. Of the non-nitrogenized articles of diet, sugar, fat, and starch, are the principal. Various inorganic substances are required to be ingested or inhaled, as water, air, chloride of sodium, iron, phosphates, lime, and sulphates. The proper amount of food containing the above elements necessary to support an average adult man, varies very much in different circumstances. Temperature is probably one of the most powerful causes of variation, as it is well known that persons living in high northern or southern latitudes will consume an amount of food sufficient for the sustenance of a large family in more temperate regions. The ingestion of alcohol, coffee, tea, tobacco, and some other substances, will, by arresting the elimination of various matters excreted from the body, retard waste, and thus enable a smaller amount of food than ordinary to support the body for a limited period of time. Probably the most correct estimate of the amount of food necessary for the sustenance of an adult, is to be found in the daily rations of the American soldier. We are told that 22 oz. of flour, 20 oz. of beef or 12 oz. of bacon, 16 oz. of potatoes (three times a week), 1½ oz. of rice, coffee ½ oz., beans 1 oz., sugar 2 oz., with a sufficient quantity of salt, and water *ad lib.*, are amply sufficient to sustain an average soldier during the most arduous campaign; and, in fact, there is no reason to doubt that in a temperate climate like ours such rations are amply sufficient. In proof of this, the excellent physical condition of our army, the amount of labor performed, the extraordinary percentage of recovery from capital surgical operations, may all be cited to show the army ration to be sufficient.

But how shall we arrive at a proper estimate of the amount of food actually required to support a sick man—one who is unable to sit up or take any active exercise at all? This is a problem more difficult of solution. In most instances the patient has no desire for food: shall we infer from this that no food is needed?—that no food would be digested if taken into the stomach? I think all will sustain me if I answer this question in the negative.

That point having been satisfactorily disposed of, the next question which naturally arises, is, *what kind* of food? How much can be given with benefit? With regard to the first question, I think the proper aliment for a patient cannot be arbitrarily prescribed; so much depends on the nature of the disease, the various organs implicated, the idiosyncrasy of the patient, and many other things that can only be determined by the closest scrutiny of the patient at the time.

As a general rule, when the anorexia is complete we may succeed in getting our patient to take small quantities of milk, beef-tea, egg-nog, and some of the lighter farinaceous articles of diet, as tapioca, arrow-root, corn-starch, etc., if we prescribe them in certain doses at regular intervals. The second query, as to how much of the above articles of food may be given with safety and benefit to the patient, it is sometimes very difficult to determine. I believe, as a rule, patients are not fed enough. If any of the secretions are abnormally increased, and rapid emaciation is destroying your patient, it will generally do to increase the amount of milk or beef-tea to a pint or two in twenty-four hours—and in such cases alcohol in some shape will generally be found useful. (In such cases I prefer good whisky to any other form of stimulus.) Of course the patient should be well supplied with fresh air by thorough ventilation of the apartment, and a sufficient amount of cool water is always necessary to the well-being of the patient, whether asked for or not. A little water should be given every hour, or oftener.

When from any cause the stomach persistently rejects the aliment offered, or any mechanical obstacle to the administration of food exists, enemas of beef tea and gruel may be used with good effect, and in some instances baths of milk, or other nutritious fluid, would add to the nourishment of the patient. Nutritive fluids might even be injected hopodermically as a *dernier ressort*, when food could not be retained by the stomach or rectum sufficient for the support of the patient. As soon as the patient begins to convalesce the food should be varied as much as possible, as no single article of diet contains all the elements necessary to a successful building up of the body. The appetite now comes to our aid, and suggests to the patient many articles of diet from which we may select an unobjectionable bill of fare.

In many instances a proper regulation of the kind and quantity of food becomes necessary as a prophylactic; for example, in tuberculosis. The patient presents all the signs which denote the existence of a scrofulous diathesis; his habitation is in a cellar, perhaps; his food, scanty in quantity, and poor in quality, is illy suited to sustain a healthy individual, much less to enable a scrofulous person to ward off the deposition of tubercle, which sooner or later generally occurs in such habits when poorly nourished. Now this person may not need medicine; in fact, no remedy would prevent his early demise so surely and pleasantly as a change of aliment. Remove him from his cellar; in its place substitute the open country; give him suitable employment, plenty of air and nourishing food, and you have succeeded in rescuing one victim from consumption—that fell destroyer of the human race, whose march has hitherto been scarcely interrupted by the best means the whole medical faculty have been able to devise.

In dyspepsia, that hydra-headed demon of disease, which seems to have chosen America as its home, and whose subjects comprise almost four-fifths of the whole population, we have an instance of what improper alimentation may accomplish, when assisted by habits of idleness and dissipation. But here, as in the former case, the great remedy is a proper use of aliments, air, food, and drink. How often do we find a patient who is reduced almost to a living skeleton, whose parchment skin, hollow eyes, fetid breath, and gloomy disposition, proclaim him a most loyal subject of King Dyspepsia; who has been treated and maltreated both by the profession and every quack in the country; who has swallowed nearly every drug known to pharmacy, without benefit—how often, I say, do we find that the only means which will restore such an individual to health, is a proper regulation of aliment. It may be that a teaspoonful of milk is as much as will be retained at once (in such an extreme case); but if only that much is retained we have room for hope; and by a judicious increase of the quantity, and a proper regulation of the necessary adjuncts, exercise and proper clothing, we may hope to restore such a patient to a normal condition of health. I have been fortunate enough to see one such extreme case, in the person of a young lady twenty-five years of age, whose normal weight was one hundred and forty pounds, and who was reduced to eighty pounds in less than a year's illness. In this case the stomach would reject nearly all the food taken, although given in quantities of one tablespoonful at a time. The cure of this case was effected by giving teaspoonful doses of new milk every half-hour, and gradually increasing the quantity as the stomach got able to bear it; in less than six months she was restored to her former weight and health, having taken but very few drugs.

It is a proper system of diet that has given to most irregular practitioners all the success they have ever obtained; without it their bubbles would soon burst, and they would be compelled to give up practice.

In view of all these facts I urge upon you the necessity of making a proper system of alimentation a study, in order that our usefulness be not in any way curtailed, and that we may have the consoling reflection that when any of our patients die, they were at least not starved to death for lack of aliment, which they were unable to ask for.—*Pacific Journal.*

